# Congenital megalourethra

**DOI:** 10.1016/j.amsu.2022.104926

**Published:** 2022-11-17

**Authors:** Turyalai Hakimi

**Affiliations:** Chief Division of Pediatric Surgery, Kabul University of Medical Science, Maiwand Teaching Hospital, Kabul, Afghanistan

**Keywords:** Case report, Megalourethra, Scaphoid, Fusiform, Urethroplasty

## Abstract

**Background:**

Congenital megalourethra is a urogenital anomaly characterized by a cystic dilatation and elongation of the penile urethra resulting from the absence and hypoplasia of the corpus spongiosum and corpus cavernosum, or anterior urethral valve. There are two clinical types: scaphoid and fusiform. Generally, the etiology is unknown, but it is thought to be a defect in mesodermal development. Fewer than 100 cases have been reported in the literature, and the exact incidence is unclear. In most cases, the surgical procedure is challenging and requires extensive reconstructive and/or replacement surgery.

**Case presentation:**

We present a 6-month-old boy suffering from a cystic dilatation of the penile urethra along with urine dribbling during micturition since birth. The patient was diagnosed with the scaphoid type of megalourethra and was operated on using reduction urethroplasty. On the 21st post-operative day, we removed the Foley catheter and followed the patient on two occasions (the 45th post-operative day and the 6th post-operative month) with excellent results.

**Conclusion:**

The anagement of megalourethra depends on the clinical type. Meticulous surgical technique, the use of fine suture materials with careful handling, and fixation of the vascularized flap are the main principles of an acceptable result. Observation of erectile function and fertility require long-term follow-up.

## Highlights

• Megalourethra is a rare urogenital anomaly involving the formation of the anterior urethra.
• The etiology is unknown, but it is believed to be a mesodermal developmental defect.
• It has two clinical types: scaphoid and fusiform.
• The treatment of the scaphoid clinical type is reduction urethroplasty, and the treatment of the fusiform clinical type ranges from sex reassignment to major phallic reconstruction.

## 1 Background

Congenital megalourethra (CM) is a rare type of functional obstruction of the lower urinary tract characterized by excessive dilation of the anterior urethra due to the absence of penile erectile tissues [1,2]. The condition was first described as congenital dilatation of the penile urethra without distal obstruction by Nesbit in 1955. He was the first to perform reduction urethroplasty [3]. Congenital megalourethra has two clinical types: scaphoid and fusiform [4]. The first prenatal case was reported in 1989 [5]. The diagnosis of megalourethra is mostly clinical, and the treatment is one or two-stage urethroplasty, depending on the patient's age at presentation and general condition. This case has been reported in line with the SCARE Criteria [6].

## 2 Case presentation

**Fig. 1:**
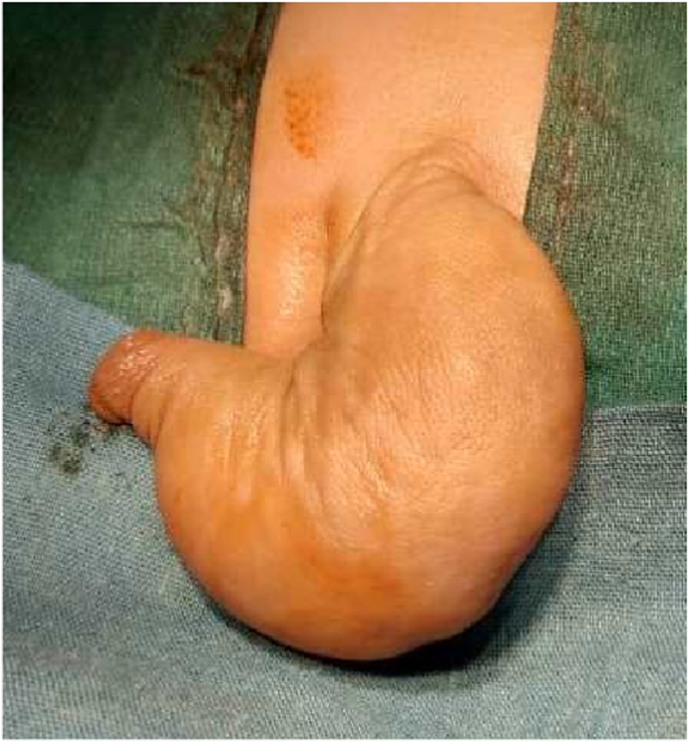
Excessive dilation of penile urethra filled with urine.

**Fig. 2:**
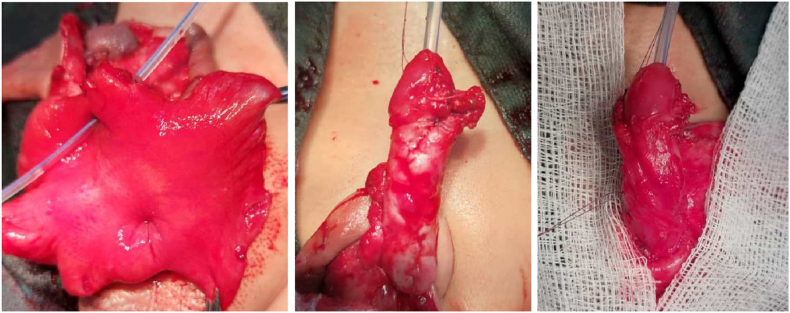
(A). Open dilated urethra following urethral midline incision with silicon catheter in place, (B). Two-layer urethroplasty, (C). Vascularized subdartos flap fixation over the urethroplasty. (For interpretation of the references to colour in this figure legend, the reader is referred to the Web version of this article.)

**Fig. 3:**
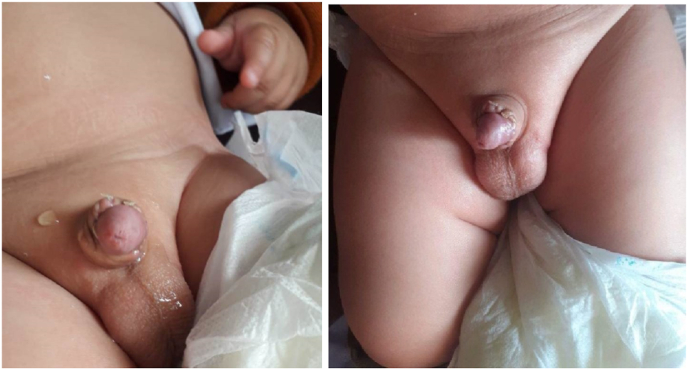
A&B Represent normal voiding and acceptable cosmetic penile appearance.

A 6-month-old boy presented with complaints of pendulous urethral dilation and urine dribbling during voiding since birth (Fig. 1). According to the parents' information, the patient was born (term and normally) to a consanguineous couple in a nearby district hospital maternity ward in the western neighboring province of the capital. All the siblings of the patient were healthy with no medical problems. Anti-natal history was unremarkable. After delivery, the attending physician noticed a disfigured penile appearance in newborn and informed the child's parents about the problem. Initially, they noticed that the penis is like a balloon, more prominent during micturition (during urinary bladder filling and tendency to micturition). The parents added that after birth, as the patient was feeling extreme pressure during voiding, they had to squeeze the patient's ballooned penile to facilitate emptying urine from the reservoir. The parents took their child to different physicians working as general practitioners (GPs) and specialists, preferably pediatricians, in the local clinic, provincial hospital, and tertiary hospital for advice and received conservative treatments. Recently, they observed marked dilatation of their child's penile urethra with inflammation of the preputial orifice, so they came to our hospital and consulted our pediatric surgery team. Upon admission, the child was examined clinically, and all routine and biochemical blood and urine tests were done. All parameters were within the normal limit, and abdominopelvic ultrasonography (USG) was also normal, but urinary tract infection (UTI) was reported. Our team treated the UTI appropriately and prepared the patient for definite treatment. We began the surgical procedure by interfering through the small preputial opening by working our way down to the penile base. During degloving, on the penile ventral aspect, we found a dilated penile urethra and a poorly developed corpus spongiosum, but an intact corpora covernosa (relatively hypoplastic), confirming the scaphoid type of megalourethra. We further mobilized the balloon urethra and excised it in the midline (Fig. 2A). The redundant urethra was excised and reduction urethroplasty was performed in three stages with an 8^fr^ silicon foley catheter as a urethral stent and 6-0 Vicryl suture. In the first stage, a subcuticular running suture was done starting from distal to proximal; in the second stage, a suture was inverted over the first plan (as reinforcement layer) (Fig. 2B); and in the third stage, a subdartos flap was harvested from the left side of the penile shaft and fixed over the entire urethroplasty suture line (Fig. 2C). Following 48 hours of post-operation, we discharged the patient and followed him up for 21 days. On the 21st day, we removed the foley catheter and followed him for 25 consecutive days onward. On the 45th post-operative day, we rechecked the patient and observed normal voiding with satisfactory cosmetic penile appearance (Fig. 3A&B). On the 6th post-operative month, the patient was re-checked with excellent result.

## 3 Discussion and conclusions

Megalourethra is a clinical condition in which penile mesodermal tissues are congenitally deficient due to faulty migration, differentiation, or development of the erectile tissue [7]. Incomplete development of erectile tissue, which normally provides support, may lead to urinary stasis and cause functional obstruction [8]. Based on the extent of erectile tissue anomalous development, megalourethra is classified into two clinical types: scaphoid and fusiform. The age of diagnosis varies between 16 weeks of gestation and 24 years [9].

Megalourethra may be associated with other genitourinary tract anomalies. Common associated anomalies are kidney dysplasia-hypoplasia, hydronephrosis, hydroureter, vesicoureteric reflux, prune-belly syndrome, urethral duplication, megacystis, hypospadias, posterior urethral valves, and cryptorchidism. Common anomalies, including VATER (vertebral, anal atresia, trachea-esophageal fistula, and renal anomalies) have also been described [10]. The workup of megalourethra includes renal function tests (RFT) and imaging of the upper and lower urinary tracts [11]. Repair of the megalourethra may require one or two-stage urethroplasty. Nesbit suggested longitudinal reduction urethroplasty (RU) for scaphoid type [3]. Heaton and colleagues described a urethral plication procedure for some cases of scaphoid megalourethra [12]. The treatment of the fusiform type ranges from sex reassignment to major phallic reconstruction [13]. Long-term follow-up is needed for the ability of erectile function to contribute to fertility [10]. Fusiform type sufferers may need placement of a penile prosthesis (PP) in adulthood [14].

Our case was a scaphoid type of megalourethra with no associated anomalies. The diagnosis was made upon delivery in the maternity hospital, and presentation for surgical repair was at the age of 6 month. Our surgical technique was reduction urethroplasty using vascularized subdartos flap (VSF) with an acceptable result at the 6th month of follow-up. Meticulous surgery along with fine suture materials and careful tissue handling (vascularized flap) are the mainstays of successful surgical result.

## Ethical approval

N/A.

## Source of funding

None.

## Author contribution

The entire process of the article from conceptualization to publication (including surgical procedure) was conducted and observed by Turyalai Hakimi.

## Consent to publish

Written informed consent was obtained from the patient parents for publication of this case report and accompanying images. A copy of the written consent is available for review by the Editor-in-Chief of this journal on request.

## Registration of research studies

1. Name of the registry: N/A.
2. Unique Identifying number or registration ID: N/A.
3. Hyperlink to your specific registration (must be publicly accessible and will be checked): N/A.

## Guarantor

Dr. Turyalai Hakimi.

## Provenance and peer review

Not commissioned, externally peer-reviewed.

## Declaration of competing interest

The author declares that he has no known competing financial interests or personal relationships that could have appeared to influence the work reported in this paper.

## List of Abbreviations

**CM**: Congenital megalourethra
**GPs**: General practitioners
**UTI**: Urinary tract infection
**USG**: Ultrasonography
**VATER**: Vertebral, anal atresia, trachea-esophageal fistula, and renal anomalies
**RU**: Reduction urethroplasty
**PP**: Penile prosthesis
**VSF**: Vascularized subdartos flap
